# Hospital admissions attributed to adverse drug reactions in tertiary care in Uganda: burden and contributing factors

**DOI:** 10.1177/20420986231188842

**Published:** 2023-07-29

**Authors:** Lillian Asio, Marble Nasasira, Ronald Kiguba

**Affiliations:** Department of Pharmacology and Therapeutics, College of Health Sciences, Makerere University, Kampala, Uganda; Child and Family Foundation Uganda, Kampala, Uganda; Department of Pharmacology and Therapeutics, College of Health Sciences, Makerere University, P.O. Box 21124, Kampala, Uganda

**Keywords:** adverse drug reaction, hospital admission, low- and middle-income countries, Sub-Saharan Africa, Uganda

## Abstract

**Background:**

Adverse drug reactions (ADRs) contribute to the burden of disease globally and of particular concern are ADR-related hospital admissions.

**Objectives:**

This study sought to determine the burden, characteristics, contributing factors and patient outcomes of ADRs that were the primary diagnosis linked to hospital admission among inpatients in Uganda.

**Design:**

We conducted a cross-sectional secondary analysis of data from a prospective cohort study of adult inpatients aged 18 years and older at Uganda’s Mulago National Referral Hospital from November 2013 to April 2014.

**Methods:**

We reviewed clinical charts to identify inpatients with an ADR as one of the admitting diagnoses and, if so, whether or not the hospital admission was primarily attributed to the ADR. Logistic regression was used to determine factors associated with hospital admissions primarily attributed to ADRs.

**Results:**

Among 762 inpatients, 14% had ADRs at hospital admission and 7% were primarily hospitalized due to ADRs. A total of 235 ADRs occurred among all inpatients and 57% of the ADRs were the primary diagnosis linked to hospital admission. The majority of ADRs occurred in people living with HIV and were attributed to antiretroviral drugs. HIV infection [aOR (adjusted odds ratio) = 2.97, 95% confidence interval (CI): 1.30–6.77], use of antiretroviral therapy (aOR = 5.46, 95% CI: 2.56–11.68), self-medication (aOR = 2.27, 95% CI: 1.14–4.55) and higher number of drugs used (aOR = 1.13, 95% CI: 1.01–1.26) were independently associated with hospital admissions attributed to ADRs.

**Conclusion:**

Antiretroviral drugs were often implicated in ADR-related hospital admissions. HIV infection (whether managed by antiretroviral therapy or not), self-medication and high pill burden were associated with hospital admissions attributable to ADRs. The high HIV burden in Sub-Saharan Africa increases the risk of ADR-related hospitalization implying the need for emphasis on early detection, monitoring and appropriate management of ADRs associated with hospital admission in people living with HIV.

## Plain language summary

Prevalence and contributing factors of hospital admissions attributed to adverse drug reactions in Uganda

**Introduction:** Adverse drug reactions (ADRs) are a big problem in many parts of the world and of particular concern is a situation where patients are admitted primarily because of an ADR. We sought to determine the burden, characteristics, contributing factors and patient outcomes of ADRs that were the primary diagnosis linked to hospital admission in Uganda.

**Methods:** We analysed data collected from a prospective cohort study of adult inpatients aged 18 years and older at Uganda’s Mulago National Referral Hospital from November 2013 to April 2014. We reviewed clinical charts to identify inpatients in whom an ADR was one of the admitting diagnoses and, if so, whether or not the ADR was the primary diagnosis linked to hospital admission. We used statistical tests to assess for contributing factors of hospital admissions attributable to ADRs.

**Results:** Among 762 inpatients, 108 had ADRs at admission and 56 were primarily admitted due to ADRs. A total of 235 ADRs occurred among all inpatients and 135 of the ADRs were the primary diagnosis linked to hospital admission. The majority of ADRs occurred in people living with HIV and were linked to antiretroviral drugs. HIV infection, use of antiretroviral therapy, self-medication and higher number of drugs used were associated with hospital admissions primarily attributed to ADRs.

**Conclusion:** To prevent hospital admissions attributed to ADRs, patients need to be warned against self-medication, health workers and patients need to be reminded of the importance of early detection, monitoring and appropriate management of ADRs among the HIV-infected (whether managed by ART or not) and those patients taking many drugs.

## Background

An adverse drug reaction (ADR) is an undesirable medical occurrence that develops after the administration of a drug at doses normally used for treatment.^1^ ADRs contribute to the burden of disease and death globally,^2,3^ of particular concern are ADR-related hospital admissions.^4–6^ About 0.2–59.6% of hospital admissions are attributable to ADRs^6–9^ and 1.8% of them are fatal in low- and middle-income countries (LMIC).^6^ The drugs most frequently implicated in high income countries (HIC) are antithrombotics (oral anticoagulants and antiplatelet agents), non-steroidal anti-inflammatory drugs and cardiovascular medications while antiretrovirals, antibacterials and antituberculosis drugs are the commonest in LMIC.^6,10^ Recent studies in Uganda among the admitted elderly implicate cardiovascular drugs, nervous system agents and anti-infective agents as the common cause of ADRs.^11,12^ Self-medication has been highlighted to be associated with ADR-related hospitalization in HIC^13,14^; however, such data are scarce in Sub-Saharan Africa (SSA) where self-medication is rampant.

Being a woman, higher number of administered drugs, comorbidities and the use of antiretroviral therapy are associated with ADR-related hospital admissions in South Africa.^15^ Studies conducted in Uganda have reported female gender and history of tobacco use as predictors of medication-related emergency admission among cardiovascular disease patients^12^; being aged 19–59 years, herbal medicine use, polypharmacy and drug-drug interactions are associated with ADRs among heart failure patients^16^; and preadmission use of herbal medicines and treatment with six or more conventional medicines during hospitalization are the common risk factors for hospital-acquired suspected ADRs.^17^ These studies document the epidemiology of hospital-acquired ADRs and ADRs in the elderly who do not represent the general population.^11,12,15–17^ Thus, the burden and associated factors of ADRs linked to hospital admission are not well investigated among inpatients in SSA, particularly in Uganda, where the risk of ADR-related hospitalizations could be heightened by the colliding epidemics of infectious and non-infectious diseases.^18^ This study sought to determine the burden, characteristics, contributing factors and patient outcomes of ADRs documented as the primary diagnosis linked to hospital admission among adult inpatients at a tertiary care hospital in Uganda.

## Methods

### Study setting and population

This cross-sectional study is a secondary analysis of data from a previously assembled prospective cohort study of adult inpatients aged 18 years and older at Uganda’s 1790-bed Mulago National Referral Hospital from November 2013 to April 2014. This tertiary care hospital is the largest and oldest government hospital providing varying inpatient and outpatient services. It is a teaching hospital and has an annual inpatient turnover of 140,000 inpatients. The study setting comprised three medical wards (Infectious Diseases and Gastrointestinal Illnesses; Haematology, Neurology and Endocrinology; Cardiovascular, Pulmonology and Nephrology) and one gynaecology ward.^17^ The study participants were aged 18 years and older and were admitted to the three medical wards and one gynaecology ward. Only one admission was considered for each inpatient. Each ward has an official bed capacity of 54 and can accommodate up to 70–80 inpatients. The medical wards average 25–40 admissions per day whilst the gynaecology ward averages 20–25 admissions per day. Study participants gave written informed consent and ethical approval for the study was obtained from the Makerere University School of Biomedical Sciences Research and Ethics Committee.^17^

### Assessment of ADR-related hospital admission

Operationally, an ADR was any undesirable medical occurrence that developed after the administration of a drug.^1^ We reviewed clinical charts to identify patients with an ADR as one of the admitting diagnoses and, if so, whether or not the ADR was the primary diagnosis linked to hospital admission. ADRs were characterized by causality, preventability, severity and outcomes. Causality was defined as at least a ‘possible’ causal relationship between the drug and the event as measured by the Naranjo ADR Probability Scale.^19^ Preventability of ADR-related hospital admission was measured by the modified Schumock and Thornton Preventability Scale.^20^ We evaluated severity using the Division of AIDS Table for Grading the Severity of Adult and Paediatric Adverse Events.^21^ Assessment of ADR-related hospital admission and outcomes were done by clinical examination. Consensus agreement on ADR causality, preventability, severity and outcomes was reached in a committee headed by the ward-based study physician and senior clinical pharmacist (principal author).^17^

### Data collection, management and analysis

We extracted variables of interest from the parent cohort study database. The extracted data included relevant baseline data on demographics, clinical conditions and medications. We categorized an ADR as whether or not it was the primary diagnosis linked to hospital admission based on the clinician’s assessment. The data were cleaned and analysed using Stata MP version 15 (StataCorp. Stata Statistical Software: Release 15. College Station, TX: StataCorp LLC). Bivalent logistic regression analysis considered a *p*-value of 0.2 to screen for variables to be included in the multivariable analyses. Categorical independent variables included HIV and antiretroviral therapy (HIV-negative/unknown, HIV-infected and on ART, HIV-infected not on ART), antituberculosis therapy (yes/no), self-medication (yes/no) and history of drug allergies (yes/no). Continuous independent variables included age, comorbidity score and number of drugs. The ADR as the primary diagnosis linked to hospital admission was the main outcome variable. Odds ratios with their 95% confidence intervals (CIs) were computed to determine the contributing factors to ADR-related hospital admission. Age in years, sex, HIV and antiretroviral therapy, antituberculosis therapy, self-medication, history of drug allergies, comorbidity score and number of drugs were evaluated in the multivariable modeling. Variables with a *p*-value of 0.05 were considered significantly associated with the outcome – ADR as the primary diagnosis linked to hospital admission.

## Results

### Characteristics of study inpatients

A total of 762 inpatients aged 18–88 years and median age of 30 years [interquartile range (IQR) of 24–42 years] were enrolled. The majority of inpatients were female (70%, 534/762) and admitted to the medical wards (75%, 571/762). More than a quarter of the inpatients were HIV-positive (30%, 232/762) and 3 in 20 (15%, 113/762) had self-medicated prior to hospital admission. A median of four drugs (IQR of 2–6 drugs) were used during the 1 month prior to hospitalization amongst inpatients with information on the number of drugs used preadmission (83%, 632/762), see Table 1.

**Table 1.** Characteristics of 762 study inpatients, Kampala, Uganda.

Characteristic	All inpatients* (*N* = 762)	Inpatients without ADRs at admission^$^ (*n* = 654)	Inpatients with ADRs at admission^$^ (*n* = 108)		
			All inpatients with ADR (*n* = 108)	Inpatients with ADR as primary diagnosis leading to admission (*n* = 56)	Inpatients in whom ADR is not the primary diagnosis of admission (*n* = 57)
Age in years, median (IQR)^‡^	30 (24–42)	30 (23–41)	33 (26–43)	35 (28–44)	30 (23–41)
Sex, *n* (%)					
Female	534 (70)	459 (86)	75 (14)	37 (7)	38 (7)
Male	228 (30)	195 (86)	33 (14)	19 (8)	14 (6)
Ward type, *n* (%)					
Medical	571 (75)	482 (84)	89 (16)	49 (9)	40 (7)
Gynaecological	191 (25)	172 (90)	19 (10)	7 (4)	12 (6)
HIV serostatus, *n* (%)					
Positive	232 (30)	170 (73)	62 (27)	35 (15)	27 (12)
Negative/unknown	530 (70)	484 (91)	46 (9)	21 (4)	25 (5)
Self-medication, *n* (%)					
Yes	113 (15)	90 (80)	23 (20)	15 (13)	8 (7)
No/unknown	649 (85)	564 (87)	85 (13)	41 (6)	44 (7)
Number of drugs, median (IQR)^§^	4 (2–6)	4 (2–6)	5 (4–7)	5 (4–7)	5.5 (4–7)
History of drug-allergy, *n* (%)					
Yes	73 (10)	55 (75)	18 (25)	10 (14)	8 (11)
No/unknown	689 (90)	599 (87)	90 (13)	46 (7)	44 (6)

Prevalence of ADR-related hospital admissions was 14% [108/762; 95% confidence interval (CI): 12–17%]; ADRs were the primary diagnosis linked to admission in 52% (56/108) of ADR-related hospitalizations; Prevalence of hospital admissions attributable to ADRs was 7% (56/762; 95% CI: 6–9%).

* [ ] represents column percentages of categorical variables except age and number of drugs which are continuous variables.

$ ( ) represents row percentages of categorical variables with the denominator as ‘All inpatients’ for that row except age and number of drugs which are continuous variables.

‡ Age range was 18–88 years.

§ Represents total number of drugs used in the 1 month period prior to hospital admission among the inpatients (83%, 632/762) who had information on the number of drugs used during the 1 month period prior to hospital admission.

ADR, adverse drug reaction; IQR, interquartile range.

### Prevalence of ADR-related hospital admission

The overall prevalence of ADR-related hospital admission was 14% (108/762; 95% CI: 12–17%) with ADRs being the documented primary diagnosis linked to hospitalization in 52% (56/108; 95% CI: 42–62%) of these admissions. Therefore, ADRs were the documented primary diagnosis linked to hospital admission in 7% (56/762; 95% CI: 6–9%) of all inpatients; and 8% (52/632; 95% CI: 6–11%) of the inpatients with detail on drug exposure during the 1 month prior to admission. Eight in thirty (27%, 62/232) HIV-positive inpatients experienced an ADR at admission with the ADR being the primary diagnosis linked to admission in 15% (35/232) of the inpatients; while 9% (46/530) of the inpatients with HIV-negative or HIV unknown status had ADR at admission with 4% (21/530) of these having an ADR as the primary diagnosis linked to hospital admission, see Table 1.

### Frequency and characteristics of ADRs related to hospital admission

A total of 235 ADRs were encountered by 108 inpatients at admission of which 57% (135/235) were the primary diagnosis linked to hospitalization for 56 of the 108 inpatients, see Table 2. Seventy two percent (97/135) were among the known HIV-infected inpatients; 38% (37/97) of these were preventable, 40% (39/97) were probable in terms of causality, 60% (56/97) had an ongoing outcome, 45% (44/97) were of moderate severity and 44% (42/97) were life-threatening, see Table 2. There were two fatal cases at hospital admission; one was shortness of breath in a HIV-negative patient and vomiting in a HIV-positive patient.

**Table 2.** Causality, preventability and severity of ADRs experienced among 108 inpatients at hospital admission, Kampala, Uganda.

Characteristic	Category	Frequency of ADRs, *n* (%)			
		All ADRs experienced at hospital admission* (*n* = 235)		ADRs that were the primary diagnosis at hospital admission^$^ (*n* = 135)	
		Known as HIV-infected (*n* = 145)	HIV-negative/unknown (*n* = 90)	Known as HIV-infected (*n* = 97)	HIV-negative/unknown (*n* = 38)
Causality	Definite	15 (10)	2 (2)	9 (9)	2 (5)
	Probable	55 (38)	24 (27)	39 (40)	12 (32)
	Possible	75 (52)	64 (71)	49 (51)	24 (63)
Preventability	Definitely or probably preventable	55 (38)	53 (59)	37 (38)	24 (63)
	Not preventable	90 (62)	37 (41)	60 (62)	14 (37)
Severity	Mild	43 (30)	27 (30)	11 (11)	2 (5)
	Moderate	60 (41)	47 (52)	44 (45)	20 (53)
	Severe or life-threatening	42 (29)	16 (18)	42 (44)	16 (42)
Outcome	Resolved	60 (41)	62 (69)	37 (38)	22 (58)
	Ongoing	83 (57)	25 (28)	58 (60)	14 (36)
	Fatal	1 (1)	1 (1)	1 (1)	1 (3)
	Unknown	1 (1)	2 (2)	1 (1)	1 (3)

* A total of 235 ADRs were experienced by 108 inpatients at hospital admission: Of these, 145 (62%) ADRs were among the known HIV-infected inpatients.

$ 135 of the 235 (57.4%) ADRs were the primary diagnosis linked to hospital admission and of these 97 (72%) ADRs were among the known HIV-infected in 56 of the 108 inpatients with ADRs at hospital admission.

ADR, adverse drug reaction.

**Figure 1.**
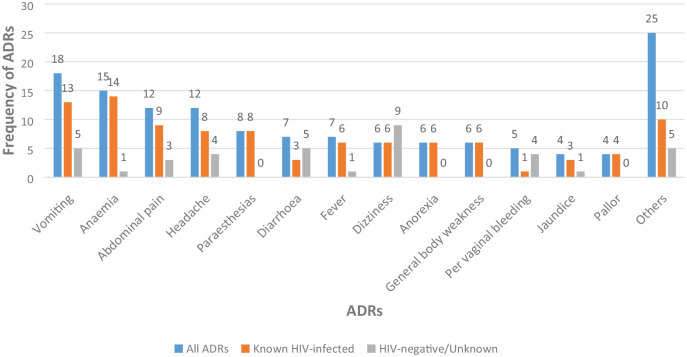
Frequency of ADRs that were the primary diagnosis linked to hospital admission among 56 inpatients, Kampala, Uganda. ADR, adverse drug reaction.

The five most common ADRs leading to hospitalization were vomiting (18 cases, 13%), anaemia (15 cases, 11%), abdominal pain (12 cases, 9%), headache (12 cases, 9%) and paraesthesias (8 cases, 6%). Vomiting, anaemia and abdominal pain were predominant in HIV-positive inpatients while dizziness, diarrhoea and vomiting were predominant in HIV-negative/unknown inpatients, see Figure 1.

Table 3 shows the drugs most commonly implicated in hospital admissions attributable to ADRs. Seventy seven percent (229/296) of the ADRs were a primary diagnosis for hospital admission and were linked to various drugs among the HIV-infected patients. The frequency of ADRs that were the primary diagnosis linked to hospital admission (*n* = 296) in the HIV-infected and HIV-uninfected/unknown serostatus inpatients is higher than the 135 ADRs reported as the primary diagnosis linked to hospital admission because some ADRs were as a result of more than one drug. Lamivudine (20%, 46/229), Tenofovir (17 %, 39/229) and Efavirenz (10%, 11/229) were the most implicated drugs at hospital admission in the HIV-infected inpatients while herbals (10%, 7/67), misoprostol (6%, 4/67), nifedipine (5%, 3/67) and ceftriaxone (5%, 3/67) were the most implicated drugs at hospital admission among the HIV-negative/unknown HIV status inpatients.

**Table 3.** Drugs most frequently implicated in ADR-related hospital admissions among 108 inpatients, Kampala, Uganda.

Implicated drugs among HIV-infected, *n* (%)			Implicated drugs among HIV-negative/unknown, *n* (%)		
	ADR present at hospital admission (*n* = 323)	ADR as the primary diagnosis linked to hospital admission^$^ (*n* = 229)		ADR present at hospital admission (*n* = 160)	ADR as the primary diagnosis linked to hospital admission^$^ (*n* = 67)
Lamivudine	53 (16)	46 (20)	Ceftriaxone	12 (8)	3 (5)
Tenofovir	43 (13)	39 (17)	Herbals	9 (6)	7 (10)
Efavirenz	33 (10)	25 (11)	Misoprostol	9 (6)	4 (6)
Zidovudine	33 (10)	23 (10)	Metro	5 (3)	2 (3)
Cotrimoxazole	26 (8)	14 (6)	Salbutamol	5 (3)	0 (0)
Rifampicin	16 (5)	12 (5)	Nifedipine	6 (4)	3 (5)
Ceftriaxone	15 (5)	6 (3)	Levofloxacin	6 (4)	0 (0)
Isoniazid	14 (4)	11 (5)	Coartem	4 (3)	2 (3)
Pyrazinamide	12 (4)	10 (4)	Cipro	4 (3)	2 (3)
Nevirapine	12 (4)	5 (2)	Frusemide	4 (3)	0 (0)
Ethambutol	10 (3)	7 (3)	Captopril	4 (3)	1 (2)
Metronidazole	7 (2)	2 (1)	Carvedilol	4 (3)	0 (0)
Nifedipine	4 (1)	3 (1)	Isoniazid	4 (3)	1 (2)
Captopril	4 (1)	4 (2)	Metformin	4 (3)	2 (3)
Frusemide	4 (1)	3 (1)	Glibenclamide	4 (3)	2 (3)
Coartem	4 (1)	1 (0)	Ethambutol	3 (2)	1 (2)
Fluconazole	4 (1)	2 (1)	Amlodipine	3 (2)	0 (0)
Others	29 (9)	16 (7)	Others	70 (44)	37 (55)

$ Some ADRs were linked to more than one drug and therefore the total frequency of ADRs (296) as the primary diagnosis linked to hospital admission is higher than total number of ADRs encountered (135).

ADR, adverse drug reaction.

Table 4 shows the frequency of ADRs linked to the 10 most implicated drugs in causing ADR-related hospital admission. Vomiting was the commonest ADR for Lamivudine (8 cases), Tenofovir (7 cases) and Efavirenz (6 cases). Severe anaemia (8 cases) was the commonest for zidovudine. Jaundice was linked to Lamivudine, Zidovudine, Isoniazid and Cotrimoxazole. Paraesthesias were linked to Lamivudine, Tenofovir, Efavirenz, Zidovudine, Isoniazid, Rifampicin, Pyrazinamide and Ethambutol.

**Table 4.** Frequency of ADRs that were the primary diagnosis linked to hospital admission for the 10 most implicated drugs in 56 inpatients, Kampala, Uganda.

Drug	ADR (*n*)
Lamivudine	Vomiting (8), Paraesthesia (7), Anaemia (5), General body weakness (5), Abdominal pain (3), Dizziness (3), Severe pallor (2), Loss of appetite (3), Fever (2), Persistent diarrhoea (2), Headache (1), Maculo-papular rash (1), Respiratory distress (1), Deep jaundice (1), Hypoglycaemia (1), Dehydration (1), Joint pain (1)
Tenofovir	Vomiting (7), General body weakness (5), Severe anaemia (4), Dizziness (3), Abdominal pain (4), Paraesthesia (2), Persistent diarrhoea (2), Loss of appetite (4), Severe pallor (2), Dehydration (1), Fever (1), Joint pain (1), Pruritus (1), Respiratory distress (1), Productive cough (1), Maculo-papular rash (1)
Efavirenz	Vomiting (6), Abdominal pain (4), Headache (3), Nightmares (2), Persistent diarrhoea (2), General body weakness (2), Anorexia (1), Hallucinations (1), Maculo-papular rash (1), Paraesthesia (1), Pruritus (1), Dehydration (1), Dizziness (1)
Zidovudine	Severe anaemia (8), Paraesthesia (4), Headache (2), Dizziness (2), Severe pallor (2), Hypoglycaemia (1), Palpitations (1), Deep jaundice (1), Fever (1), General body weakness (1)
Cotrimoxazole	Severe anaemia (3), Generalized maculo-papular rash (1), Anorexia (2), Diarrhoea (2), Vomiting (1), Joint pain (1), Worsened pallor (1), Headache (1), Paraesthesia (1), Jaundice (1)
Ceftriaxone	Vomiting (4), Fever (3), Worsened jaundice (1)
Isoniazid	Deep jaundice (2), Vomiting (2), Peripheral neuropathy (2), Fever (1), Abdominal pain (1), Nausea (1), Dizziness (1), Loss of appetite (1), Pruritus (1), Shortness of breath (1)
Rifampicin	Vomiting (3), Paraesthesia (1), Deep jaundice (1), Dizziness (1), Abdominal pain (1), Pedal oedema (1), Shortness of breath (1), General body weakness (1), Nausea (1), Loss of appetite (1)
Pyrazinamide	Vomiting (2), Deep jaundice (2), Paraesthesia (1), Abdominal pain (1), Fever (1), Loss of appetite (1), Nausea (1), Dizziness (1), Shortness of breath (1)
Ethambutol	Vomiting (2), Paraesthesia (2), Shortness of breath (1), Dizziness (1), Pruritus (1), Nausea (1), Abdominal pain (1)

ADR, adverse drug reaction.

### Factors associated with hospital admissions attributable to ADRs

Table 5 shows that HIV infection (aOR (adjusted odds ratio) = 2.97, 95% CI: 1.30–6.77), use of antiretroviral therapy (aOR = 5.46, 95% CI: 2.56–11.68), self-medication during the 1 month prior to admission (aOR = 2.27, 95% CI: 1.14–4.55) and higher number of drugs used in the 1 month prior to admission (aOR = 1.13, 95% CI: 1.01–1.26) were independently associated with a higher likelihood for hospital admissions attributable to ADRs.

**Table 5.** Factors associated with hospital admissions attributable to ADRs in 632 inpatients with information on the number of drugs used during the 1 month before admission, Uganda.

Variable	ADR as the primary diagnosis linked to hospital admission		Crude analysis			Adjusted analysis		
	Yes (*n* = 52)	No (*n* = 580)	cOR	95% CI	*p*-Value	aOR	95% CI	*p*-Value
Age in years, mean (*SD*)	37.38 (11.69)	35.65 (15.14)	1.01	0.99–1.03	0.422	1.00	0.98–1.03	0.881
Sex, *n* (%)								
Male	19 (10)	180 (90)	1.00			1.00		
Female	33 (8)	400 (92)	0.78	0.43–1.41	0.414	0.74	0.40–1.40	0.358
HIV and antiretroviral therapy, *n* (%)								
HIV-negative/unknown	17 (4)	392 (96)	1.00			1.00		
HIV-infected, not on ART	11 (11)	91 (89)	2.79	1.26–6.15	0.011	2.97	1.30–6.77	0.010
HIV-infected, on ART	24 (20)	97 (80)	5.71	2.95–11.04	<0.001	5.46	2.56–11.68	<0.001
Antituberculosis therapy, *n* (%)								
No	47 (8)	539 (92)	1.00			1.00		
Yes	5 (11)	41 (89)	1.40	0.53–3.71	0.500	0.58	0.20–1.69	0.321
Self-medication, *n* (%)								
No	37 (7)	488 (93)	1.00			1.00		
Yes	15 (14)	92 (86)	2.13	1.14–4.00	0.019	2.27	1.14–4.55	0.020
History of drug allergies, *n* (%)								
No/unknown	42 (7)	524 (93)	1.00			1.00		
Yes	10 (15)	56 (85)	2.23	1.06–4.68	0.035	1.78	0.80–3.95	0.155
Comorbidity score, mean (*SD*)	0.77 (1.29)	0.76 (1.23)	1.00	0.80–1.26	0.968	1.11	0.81–1.52	0.521
Number of drugs, mean (*SD*)	5.92 (2.85)	4.32 (2.64)	1.21	1.10–1.33	<0.001	1.13	1.01–1.26	0.029

ADR, adverse drug reaction; aOR, adjusted odds ratio; CI, confidence interval; cOR, crude odds ratio; *SD*, standard deviation.

### Patient outcomes

In-hospital mortality attributable to ADRs occurred in two inpatients (0.3%, 2/762; 95% CI: 0–0.9%). In-hospital all-cause mortality among inpatients with an ADR as the primary diagnosis linked to admission was similar to in-hospital all-cause mortality among inpatients admitted for other reasons [4% (2/56; 95% CI: 0–12%) *versus* 4% (30/706; 95% CI: 3–6%), respectively; *p* = 1.000].

The median length of hospital stay for all inpatients was 5 days (IQR: 3–6 days) and was similar among inpatients admitted for ADRs (median of 5 days; IQR of 3–6 days) *versus* those admitted for other reasons (median of 5 days; IQR of 3–6 days).

## Discussion

In this cross-sectional study conducted among 762 inpatients at Mulago National Referral Hospital, 14% had ADRs at admission and 7% were primarily admitted due to ADRs. A total of 235 ADRs occurred among all inpatients and 135 ADRs among those primarily admitted due to ADRs, predominantly vomiting (13%) and anaemia (11%). The majority of ADRs occurred in HIV-infected inpatients and were attributed to antiretroviral drugs such as Lamivudine, Tenofovir, Efavirenz and Zidovudine. Severe or life-threatening ADRs constituted 29% of all ADRs and 38% of ADRs among the known HIV-infected inpatients were preventable. HIV infection (whether managed by ART or not), self-medication and high pill burden were associated with hospital admission attributable to ADRs. Mortality and length of hospital stay were the same irrespective of the primary reason of admission (ADRs or other conditions). These findings show that community-acquired ADRs are an important contributor to morbidity in Uganda; however, most of the contributing factors are modifiable if there is integrated individualized care for these special groups.

One in seven admitted patients had an ADR and 7% of the admissions had an ADR as the primary diagnosis. This is consistent with findings from other studies which reported a prevalence of 1.1–16.9%.^6,10,15,22^ This is because tertiary institutions in high HIV/AIDS burden countries admit many patients with HIV and ADRs are majorly attributable to the ART. Earlier ART regimens containing Zidovudine or Efavirenz caused more ADRs than the newer regimens containing Dolutegravir. With increased rollout of the more tolerable Dolutegravir-based ART regimens, ADRs at admission could substantially decrease. Additionally, lower HIV prevalence and ART use will further lower ADRs at admission.^23^ However, even with the expected drop in ADR-related admissions, health workers should routinely screen for suspected toxicities in individuals on ART to minimize ADR-related hospital admissions. ADRs were the primary diagnosis linked to admissions in only 4% (21/530) of the HIV-negative inpatients. This is notably lower compared with the 15% reported in a systematic review of the literature in LMIC. This difference is explained by the age variation whereby the review included articles in predominantly elderly patients (with comorbidities such as cancer, cardiac disease and dementia) who are likely to have a cocktail of drugs, increasing the risk of hospital admission^24^ compared with our study where the average age of patients was 30 years.

People living with HIV are more likely to have ADRs at admission. Overall, 62% of all ADRs among all inpatients (72% of ADRs among those primarily admitted due to ADRs) occurred among people living with HIV (PLHIV). The PLHIV not on ART were thrice as likely to be hospitalized due to ADRs as compared with inpatients without HIV or of unknown HIV status. Co-medications for opportunistic infections and other HIV/AIDS comorbidities could have contributed to the ADRs among PLHIV not on ART.^25^ This reinforces the Uganda HIV guidelines which state that clinicians should provide information to patients on ADRs of the drugs for management of opportunistic infections. Patients on ART are five times more likely to be hospitalized due to ADRs as compared with inpatients without HIV or of unknown HIV status. This higher rate of ADRs is attributable to ART regimens, for example, Lamivudine (16%), Tenofovir (13%) and Efavirenz (10%) which were the most implicated drugs among the HIV-positive inpatients. This is similar to findings reported by a systematic review which reported immunosuppression with HIV and ART as an increased risk for ADR-related hospitalization in developing countries.^6^ However, ADR-related admissions due to ART are likely to decrease with the fall in HIV prevalence and use of more tolerable Dolutegravir regimens. Note that Lamivudine and Tenofovir, the top two ADR-implicated drugs, are components of the newer ART regimens in LMIC.

Ceftriaxone was the most reported drug to be used prior to hospital admission among patients presenting with ADRs at admission while herbals, misoprostol, nifedipine and ceftriaxone were the most implicated drugs linked to hospital admission among HIV-negative or unknown HIV status inpatients. These findings are unique to our study population given that gynaecological patients were included in this study. Other studies among admitted elderly patients have reported cardiovascular drugs, nervous system agents and anti-infective agents as the cause of ADRs.^11,12^ A study in Uganda by Kiguba et al.^17^ reported herbal medicines to be linked to hospital-acquired ADRs. The Uganda treatment guidelines require that misoprostol is administered from a hospital setting; however, it is a common practice for patients to self-medicate with misoprostol to initiate abortion before seeking professional care which causes adverse events such as incomplete abortion, failed abortion, missed abortion, severe anaemia, haemorrhagic shock, sepsis, ectopic pregnancy and ruptured uterus^26^ requiring hospital admissions.

Inpatients who self-medicated or used multiple drugs were more likely to be hospitalized due to ADRs, which is consistent with the literature.^4,27^ Self-medication is a known risk factor for ADRs.^14,27^ Some ADRs are preventable with clinician and pharmacist prescription fidelity, unlike self-medication. Polypharmacy and the use of multiple drugs increases the risk of drug-drug interactions, some of which could be serious, requiring hospitalization.^28^ Patient education on the negative effects of self-medication and regulatory restrictions to classified medicines could help reduce self-medication and consequently curb admissions linked to ADRs. Pharmacists are critical in examining complex multiple treatment regimens for inpatients, such as treatments for HIV and comorbidities and advising clinical teams on revising regimens to reduce drug-interactions and ADR-related admissions.^29^

This study has important limitations. We assessed ADRs based on clinical judgment and were not able to do laboratory investigations, which could underreport the ADR-related hospital admissions. Some of the associated factors such as history of drug use were based on patient self-report which could have introduced recall bias, possibly leading to increased or decreased strength of the observed associations. Given that this was a cross-sectional study, we were not able to fully establish the causal association of ADRs and hospital admissions. However, we reviewed admissions where ADRs were recorded by the clinicians as being the primary diagnosis linked to hospital admission, more than just ADRs being present at admission. The study findings are limited to the gynaecological and medical settings and might not be generalizable to surgical and paediatric wards. We conducted the study in a single tertiary care hospital that serves referred patients with severe illnesses and a higher number of comorbidities, which makes the findings slightly more difficult to generalize to other patient populations.

## Conclusion

Antiretroviral and antituberculosis drugs were often implicated in ADR-related hospital admission. HIV infection (whether managed by ART or not), self-medication and high pill burden were associated with hospital admission attributable to ADRs. The high HIV burden in SSA increases the risk of ADR-related hospitalization implying the need to establish and strengthen systems, such as medication reconciliation, to promote the early detection, monitoring and appropriate management of ADRs linked to hospital admission among HIV-infected people in these settings.

## Supplemental Material

sj-docx-1-taw-10.1177_20420986231188842 – Supplemental material for Hospital admissions attributed to adverse drug reactions in tertiary care in Uganda: burden and contributing factors Click here for additional data file. Supplemental material, sj-docx-1-taw-10.1177_20420986231188842 for Hospital admissions attributed to adverse drug reactions in tertiary care in Uganda: burden and contributing factors by Lillian Asio, Marble Nasasira and Ronald Kiguba in Therapeutic Advances in Drug Safety

## Declarations

**Ethics approval and consent to participate:** Ethical approval for this study study was obtained from the School of Medicine Research and Ethics Committee, Makerere University College of Health Sciences (REC REF No. 2011–113), the Mulago Hospital Research and Ethics Committee (MREC 253) and the Uganda National Council for Science and Technology (HS 1151). Voluntary participation of the inpatients (⩾18 years) was sought through provision of written informed consent.

**Consent for publication:** Not applicable.

**Availability of data and materials:** The data used for this paper are included in the main paper or as additional supporting files.
