# Supplementary material for: Hospital admissions attributed to adverse drug reactions in tertiary care in Uganda: burden and contributing factors
Source: Ther Adv Drug Saf. 2023 Jul 29;14:20420986231188842. doi: 10.1177/20420986231188842 (PMC10387768; doi:10.1177/20420986231188842)
Supplement: sj-docx-1-taw-10.1177_20420986231188842 – Supplemental material for Hospital admissions attributed to adverse drug reactions in tertiary care in Uganda: burden and contributing factors [file sj-docx-1-taw-10.1177_20420986231188842.docx]

**Supplementary material**

**Table 1: Frequency of ADRs that were the primary diagnosis linked to hospital admission among 56 inpatients, Kampala, Uganda**

| ADR | | Frequency of ADRs | | | | |
| --- | --- | --- | --- | --- | --- | --- |
|  | **All ADRs (n=135)** | | **Known HIV-infected (n=97)** | | **HIV-negative/Unknown (n=38)** | |
| Vomiting | | 18 (13) | | 13 (13) | | 5 (13) |
| Anaemia | | 15 (11) | | 14 (14) | | 1 (3) |
| Abdominal pain | | 12 (9) | | 9 (9) | | 3 (8) |
| Headache | | 12 (9) | | 8 (8) | | 4 (11) |
| Paraesthesias | | 8 (6) | | 8 (8) | | 0 (00) |
| Diarrhoea | | 7 (5) | | 3 (3) | | 5 (13) |
| Fever | | 7 (5) | | 6 (6) | | 1 (3) |
| Dizziness | | 6 (4) | | 6 (6) | | 9 (24) |
| Anorexia | | 6 (4) | | 6 (6) | | 0 (00) |
| General body weakness | | 6 (4) | | 6 (6) | | 0 (00) |
| Per vaginal bleeding | | 5 (4) | | 1 (1) | | 4 (11) |
| Jaundice | | 4 (3) | | 3 (3) | | 1 (3) |
| Pallor | | 4 (3) | | 4 (4) | | 0 (00) |
| Others | | 25 (19) | | 10 (10) | | 5 (13) |
| ADR is adverse drug reaction | | | | | |  |
